# Ultra‐Hypofractionated Radiotherapy for Early‐Stage Glottic Cancer: Real‐World Data From a Single Center

**DOI:** 10.1002/hed.28139

**Published:** 2025-04-01

**Authors:** Mete Gundog, Esin Kiraz

**Affiliations:** ^1^ Department of Radiation Oncology Erciyes University Kayseri Turkey

**Keywords:** laryngeal cancer, local control, radionecrosis, ultra‐hypofraction, voice quality

## Abstract

**Background:**

In recent years, smaller‐volume radiotherapy has attracted attention. It appears to be a safe and effective treatment for glottic laryngeal cancer. This study evaluated the results of ultra‐hypofractionated radiotherapy for early glottic laryngeal cancer in unfit patients.

**Methods:**

This retrospective study analyzed 22 patients diagnosed with early glottic cancer between June 2017 and January 2021. The involved vocal cord was treated with 35–42.5 Gy in 5 fractions.

**Results:**

On a 59‐month median follow‐up, the 5‐year overall survival and local control rate were 68.2% and 94.7%, respectively. However, the 5‐year larynx preservation rate was 89.7%. Chondronecrosis was observed in one patient (4.5%), and soft tissue necrosis in one patient (4.5%). PTV_D98_ (> 41.08 Gy) was found to be statistically significant in surgery‐needed events (HR: 22.4, 95% CI: 1.9–252.1, *p* = 0.01).

**Conclusion:**

Single‐cord ultra‐hypofractionated radiotherapy appears to be an effective treatment for local control. However, the risk of radionecrosis is higher at doses above 41.08 Gy.

## 1 Introduction

Laryngeal cancer is the most common non‐cutaneous head and neck cancer [1]. At the time of diagnosis, over half of cases are found in the glottic region at an early stage (T in situ or T1–T2 tumors without nodal involvement). Since the risk of nodal spread for early‐stage glottic laryngeal cancer is low, the goal is to achieve effective primary local control (LC) with a single treatment modality. Radiotherapy (RT) and transoral laser microsurgery (TLM) are the most commonly preferred treatment options. Both methods show good survival outcomes, so preserving organ function and voice quality is essential as much as possible. Studies have shown that moderate hypofractionated treatment regimens are effective and safe for whole larynx irradiation [2, 3]. Additionally, treating only the involved vocal cord reduces the irradiated volume, thus reducing the dose to the surrounding tissue. Recent studies have shown that an ultra‐hypofractionated (UHRT) approach for treating a single vocal cord can reduce treatment duration and the dose to organs at risk (OAR) [4, 5]. Reduced treatment duration can also make RT a better alternative than TLM, especially for elderly patients, those with comorbidities, contraindications to anesthesia, or individuals who lack social support and find it difficult to visit the hospital [6].

This retrospective study aims to evaluate the oncological outcomes and voice quality in patients who underwent ultra‐hypofractionation treatment of the single vocal cord area and could not receive conventional therapies due to poor general health status, medical inoperability, and limited social and logistic support. The study also aims to investigate the radiation doses received by the subunits of the larynx (ipsilateral and contralateral arytenoid, thyroid cartilage, cricoid cartilage) and the OAR (ipsilateral and contralateral carotid artery, inferior constrictor muscle, spinal cord, skin).

## 2 Materials and Methods

### 2.1 Patients

A total of 22 patients diagnosed with early‐stage glottic squamous cell cancer (SCC) (cTis‐T2N0M0 according to the 8th edition of the American Joint Committee on Cancer) between June 2017 and January 2021 were evaluated. These patients could not undergo TLM or conventional RT for the following reasons: Patients who had concomitant diseases that constituted absolute or relative contraindications to anesthesia and could not reach the hospital every day for 5–6 weeks due to lack of logistic or social support were evaluated. Patients who received second‐line radiotherapy or patients with concurrent malignant tumors were not included in this study. The procedure was started after the patients were informed about the potential side effects of the treatment.

Patient‐reported voice quality (such as best, better, same, worse, and worst) and adverse events were collected from patient records and the hospital information system. Voice quality was scored on a scale of 10–50, where lower scores indicated better voice quality. Toxicities were graded using the Common Terminology Criteria for Adverse Events version 4.0. The Institutional Clinical Research Ethics Committee (2024/90) approved the study.

### 2.2 Treatment

In all patients, contrast‐enhanced simulation tomography with a 1‐mm‐thick slice was performed with the patient in a supine position and with the head hyperextended. A thermoplastic mask covering the head, neck area, and shoulders had been obtained. None of the patients received a bolus. A 4D simulation was optional.

**FIGURE 1:**
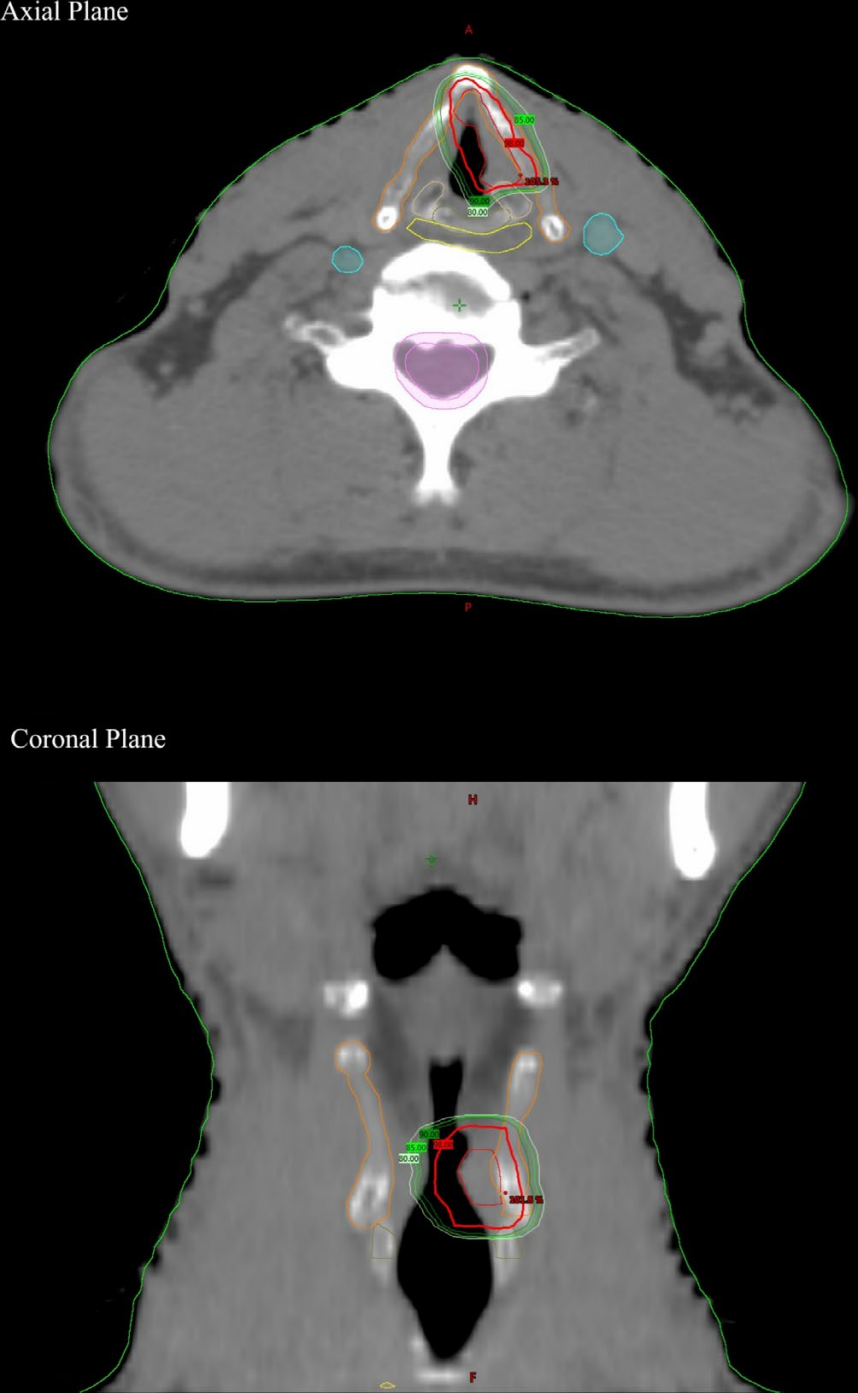
Isodose curves and structures. [Color figure can be viewed at wileyonlinelibrary.com]

The clinical target volume (CTV) included gross disease on the vocal cord. Half of the ipsilateral arytenoid cartilage was electively treated for tumors of the posterior vocal cord. Besides that, the anterior commissure was treated for tumors of the anterior vocal cord, and the anterior 2 mm of the contralateral vocal cord was treated for tumors with anterior commissure involvement. The intra‐laryngeal air space was not included. Anisotropic margins were applied for the planning target volume (PTV), with 5 mm in the cranial–caudal direction and 3 mm in the left–right and anterior–posterior directions (Figure 1). Laryngeal subunits included the ipsilateral and contralateral arytenoid, thyroid cartilage, and cricoid cartilage. The OARs included the ipsilateral and contralateral carotid artery, inferior constrictor muscle, spinal cord, and skin. Before each fraction, cone beam computed tomography (CBCT) was performed, and the attending physician approved all of them. Eclipse version 13.6 (Varian Medical System, Palo Alto, CA, USA) was used for treatment planning.

### 2.3 Follow‐Up

The established follow‐up protocol for patients diagnosed with glottic laryngeal cancer includes endoscopic examinations every 3 months for the first 2 years, every 6 months from the second to the fifth year, and then annually thereafter. CT scans are performed annually or if suspicious findings are found during an examination. This follow‐up plan was recommended for patients who were able to attend the check‐ups. The national health system was used to check the survival status and images of the head and neck area of patients who did not come to our center for follow‐up. These patients were also called by phone to evaluate complaints and voice quality.

### 2.4 Statistics

Overall survival, local control, radionecrosis rate, and larynx preserve rate were calculated using the Kaplan–Meier method. Factors influencing surgery‐needed events and biopsy‐needed events were evaluated using univariate analysis. The cut‐off value for PTV_D98_ to predict surgery and/or biopsy‐needed events was determined by receiver operating characteristic (ROC) curve analysis. The Friedman test was used to show the difference between more than two dependent variables to evaluate voice quality. The *p* values below 0.05 were considered statistically significant unless otherwise stated. All statistical analyses were performed using SPSS version 27.0 (IBM, Armonk, NY, USA) and MedCalc Software Ltd. Version 22.023.

## 3 Results

All the patients in the study were male, with a median age of 64.5 years. Among the patients, 2 (9.1%) had stage Tis, 15 (68.2%) had stage T1a, and 5 (22.7%) had stage T2. Furthermore, 15 patients (68.2%) had anterior commissure involvement. Almost all patients had a history of smoking except for two patients. The general characteristics of the patients are summarized in Table 1.

**TABLE 1:** The general characteristics of the patients.

Characteristic	*n* (%) or median	
Age (years)		
Median (IQR)	64.5	(59–69)
Minimum–maximum	51–87	
Gender		
Male	22	(100%)
Smoking status		
None	2	(9%)
Previous	7	(32%)
Active	13	(59%)
Smoking (pack per day × years)		
Median (IQR)	45	(30–62.5)
T stage		
Tis	2	(9%)
T1a	15	(68%)
T1b	0	
T2	5	(23%)
Anterior commissure		
Involved	15	(68%)
Not involved	7	(32%)
Side		
Right	11	(50%)
Left	11	(50%)
Treatment regimen		
35 Gy/5 fractions	1	(5%)
40 Gy/5 fractions	18	(82%)
42.5 Gy/5 fractions	3	(14%)
Fractionation		
2–3 fractions/week	15	(68%)
5 fractions/week	7	(32%)
Comorbidity		
Yes	16	(73%)
Cardiovascular	12	
Neuropsychiatric	3	
Kidney	2	
Pulmonary	3	
Musculoskeletal	2	
Diabetes Mellitus and Gastrointestinal	7	
No	6	(27%)

The median PTV was 6.1 cc (interquartile range [IQR]: 5.2–9.0). Four‐dimensional (4D) imaging was performed in three patients (13.6%). The median PTVs of the 4D and standard planning CT groups were 7.0 cc (IQR: 6.6–9.1) and 5.8 cc (IQR: 5.2–8.2), respectively (Mann–Whitney *U*, *p* = 0.20). The prescribed dose for the PTV was 35–42.5 Gy delivered in 5 fractions. Specifically, one patient (4.5%) received 35 Gy because he had the largest PTV and the highest smoking history (100 packs/year), 18 patients (81.8%) received 40 Gy, and three patients (13.6%) received 42.5 Gy in 5 fractions. Treatment schedules included every other day for 15 patients (68.2%) and 5 days a week for seven patients (31.8%). The median prescribed dose to 98% of the planning target volume (PTV_D98_) was 39.7 Gy (IQR: 39.5–39.9). The median dose delivered to the whole larynx was 19.8 Gy (IQR: 17.8–21.7) (Table 2). RT was administered using intensity‐modulated radiation therapy (IMRT) in three patients (13.6%) and volumetric‐modulated arc therapy (VMAT) in 19 patients (86.4%) with a 6 MV photon beam in flattening filter‐free mode from a Varian TrueBeam‐STX linear accelerator.

**TABLE 2:** Dosimetry metrics.

Organ‐at‐risk	All patients, median (IQR) (Gy), *n* = 22	35 Gy/5 fx (Gy), *n* = 1	40 Gy/5 fx, median (IQR) (Gy), *n* = 18	42.5 Gy/5 fx, median (Gy), *n* = 3
PTV volume (cc)	6.1 (5.2–9.0)	11.9	6.1 (5.2–7.9)	5.6
PTV D98	39.7 (39.5–39.9)	35.4	39.7 (39.5–39.9)	42.2
IL arytenoid max	42.2 (40.3–43.5)	36.3	42.1 (40.3–42.4)	45.1
IL arytenoid mean	38.2 (29.9–39.7)	36.0	38.2 (28.9–39.4)	43.3
CL arytenoid max	25.9 (20.9–29.0)	32.2	25.2 (20.8–27.3)	29.0
CL arytenoid mean	19.3 (17.0–21.3)	28.2	18.0 (16.6–21.0)	21.1
Cricoid cartilage max	42.8 (42.4–44.0)	36.9	42.6 (42.4–43.8)	44.7
Cricoid cartilage mean	21.4 (19.3–26.3)	28.6	21.0 (17.7–24.5)	27.5
Thyroid cartilage max	43.3 (42.5–44.4)	27.0	43.0 (42.5–44.0)	45.8
Thyroid cartilage mean	22.7 (20.1–26.7)	26.7	22.7 (20.1–26.7)	20.7
Whole larynx mean	19.8 (17.8–21.7)	26.7	19.6 (17.8–21.3)	19.7
Constrictor muscle max	31.7 (26.7–38.0)	37.0	31.2 (26.7–35.6)	41.1
Constrictor muscle mean	12.2 (9.4–13.7)	21.9	11.5 (9.2–13.3)	13.3
Esophagus max	2.5 (1.4–9.3)	4.3	3.4 (1.6–14.2)	1.3
Thyroid gland mean	1.9 (1.3–2.6)	1.6	1.9 (1.3–3.1)	1.9
IL carotid max	16.9 (12.4–18.5)	20.0	16.4 (11.7–17.8)	19.8
IL carotid mean	5.1 (4.0–6.4)	6.1	4.8 (3.8–6.0)	6.6
CL carotid max	12.6 (9.6–15.1)	15.9	12.2 (9.3–13.9)	13.8
CL carotid mean	4.0 (3.2–5.4)	5.7	3.6 (3.1–5.2)	4.8
Spinal cord max	9.1 (8.1–10.5)	8.8	9.5 (8.1–11.0)	8.3
Skin max	39.6 (31.4–41.8)	31.0	39.6 (31.3–41.5)	44.9

Abbreviations: CL, contralateral; fx, fractions; IL, ipsilateral; max, maximum.

### 3.1 Oncological Outcomes

**FIGURE 2:**
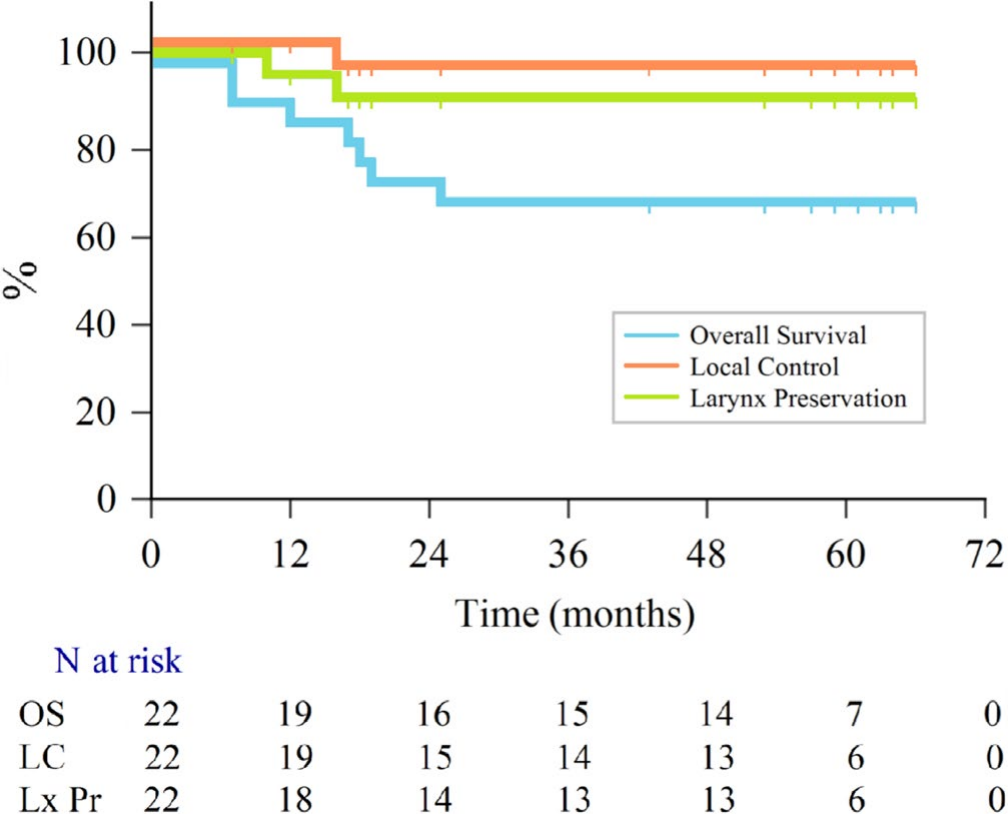
Overall survival, local control, and larynx preservation Kaplan–Meier curve. [Color figure can be viewed at wileyonlinelibrary.com]

The median follow‐up was 59 months (IQR: 57–63). During the follow‐up, five patients developed secondary lung cancer (one with small cell lung cancer and four with non‐small cell lung cancer). The 5‐year OS rate was 68.2% (Figure 2). Upon analysis, it was found that four patients had died due to cardiac‐related causes, one due to thromboembolism, and two due to secondary lung cancer. There were no laryngeal cancer‐related deaths. No lymph node in the neck or distant metastasis was detected in any patient during follow‐up. The 5‐year LC rate was 94.7%, and the 5‐year actual LC rate was 100% with the salvage TL. However, the 5‐year larynx preservation rate was 89.7% (Figure 2).

Five (22.7%) patients underwent additional examinations during the follow‐up period, but the other patients did not require any additional examinations other than routine examinations. None of the patients developed in‐field failure, while one intra‐laryngeal failure was observed in the subglottic region in the sixteenth month. This patient, who had a total laryngectomy (TL) (90% soft tissue necrosis and 10% SCC in the removed tissue), lives without the disease. Another patient underwent a biopsy 9 months after treatment because imaging results indicated a possible recurrence. The biopsy revealed SCC, and the patient underwent TL. However, no tumor tissue was found in the postoperative specimen; only chondronecrosis and fibrotic changes were present. In another patient, local excision was performed 10 months after treatment because submucosal edema was observed during follow‐up. No tumoral tissue was found in the suspicious area or other areas (Appendix S1).

So, one patient (4.5%) had cartilage necrosis, and another patient (4.5%) had soft tissue necrosis. These patients received a total dose of 42.5 Gy in 5 fractions. No event was observed in the third patient who received 42.5 Gy/5 fractions; however, this patient received a D_98_ dose of 39.7 Gy, which is comparable to the 40 Gy in the 5‐fraction group. The other two patients D98 doses were 42.5 and 42.2 Gy, respectively. The 5‐year radionecrosis‐free rate was found to be 89.7%. In these patients who underwent surgical intervention due to the risk of relapse during necrosis development, hyperbaric oxygen or steroid therapy was not administered.

Patients who underwent surgical procedures such as laryngectomy or cordectomy were considered a “surgery‐needed event” even if there was no true recurrence.

The ROC analysis indicated a statistically significant PTV D_98_ cut‐off dose associated with the surgery‐needed event was 41.08 Gy (AUC: 0.860, *p* < 0.01). Thirteen patients were still active smokers (all of them > 30 packs/year), seven were former smokers (all of them > 20 packs/year), and two patients had never smoked. Smoking status did not affect the surgery‐needed event in the cox‐regression analysis (HR: 0.01, 95% CI: 0–142.8, *p* = 0.34) (Table 3). Fifteen patients had anterior commissure involvement. Although the anterior part of the opposite cord was also irradiated in these patients, there was no significant relationship with the surgery‐needed event (HR: 0.9, 95% CI: 0.8–10.7, *p* = 0.98). In the univariate analysis, only the PTV D_98_ (< 41.08 vs. > 41.08) showed a statistically significant association with surgery‐needed events (HR: 22.4, 95% CI: 1.9–252.1, *p* = 0.01) (Table 3).

**TABLE 3:** Cox‐regression analysis for radionecrosis and larynx preservation according to clinical factors and dose‐volume.

	Time to surgery‐needed event			Time to biopsy‐needed event		
	HR	95% CI	*p*	HR	95% CI	*p*
PTV_D98_ (Gy) (< 41.08 vs. > 41.08)	22.4	1.9–252.1	0.01	6.49	1.0–39.2	0.04
PTV (cc) (< 10 cc vs. > 10 cc)	3.9	0.3–45.4	0.27	1.6	0.1–14.8	0.66
IL Arytenoid max	1.3	0.7–2.3	0.34	2.4	0.8–7.1	0.10
IL Arytenoid mean	1.3	0.9–2.0	0.19	2.6	0.7–9.7	0.16
CL Arytenoid max	1.0	0.9–1.2	0.70	1.1	0.9–1.3	0.42
CL Arytenoid mean	1.0	0.9–1.2	0.74	1.1	0.9–1.3	0.48
Cricoid max	1.3	0.6–2.9	0.49	2.3	0.6–8.6	0.22
Cricoid mean	1.1	1.0–1.2	0.20	0.1	0–34.7	0.44
Constrictor max	1.1	0.9–1.3	0.39	1.9	0.6–6.1	0.26
Constrictor mean	1.1	1.0–1.3	0.11	0.1	0–34.9	0.46
Smoke status (non‐active vs active)	0.01	0–142.8	0.34	0.1	0–1.4	0.10
Anterior Commissure (involved vs. uninvolved)	0.9	0.8–10.7	0.98	2.1	0.2–19.3	0.49
T stage (Tis/T1a vs. T2)	2.2	0.2–25.1	0.52	0.7	0.1–4.7	0.67
Fractionation (5 fx/week vs. 3 fx/week)	1.2	0.1–13.7	0.85	2.5	0.2–39.4	0.52

Abbreviations: cc, cubic centimeter; CL, contralateral; fx, fractions; Gy, gray; IL, ipsilateral; PTV, planning target volume.

### 3.2 Toxicity

No patients experienced toxicity during radiation therapy that required treatment interruption. Acute toxicity did not develop in 15 patients (68.2%), and in 10 of these patients (45.5%), late toxicity also did not occur. Late G3 laryngitis (actinomyces infection) developed in one patient (4.5%) who underwent TL because of suspected recurrence after 16 months. No acute or late G3 or higher toxicity was observed apart from this. The most frequently observed acute toxicity was laryngeal edema (*n* = 3, 13.6%) and dysphagia (*n* = 3, 13.6%). G1–2 laryngeal edema was the most commonly observed late toxicity (*n* = 6, 27.3%) (Table 4).

**TABLE 4:** Treatment toxicity.

	Acute toxicity		Late toxicity		
	Grade 1	Grade 2	Grade 1	Grade 2	Grade 3
Laryngeal edema	3 (14%)	—	3 (14%)	3 (14%)	—
Hoarseness	2 (9%)	—	2 (9%)	1 (5%)	—
Dysphagia	1 (5%)	2 (9%)	—	—	—
Couch	—	1 (5%)	1 (5%)	—	—
Laryngitis	—	—	—	1 (5%)	1 (5%)

**FIGURE 3:**
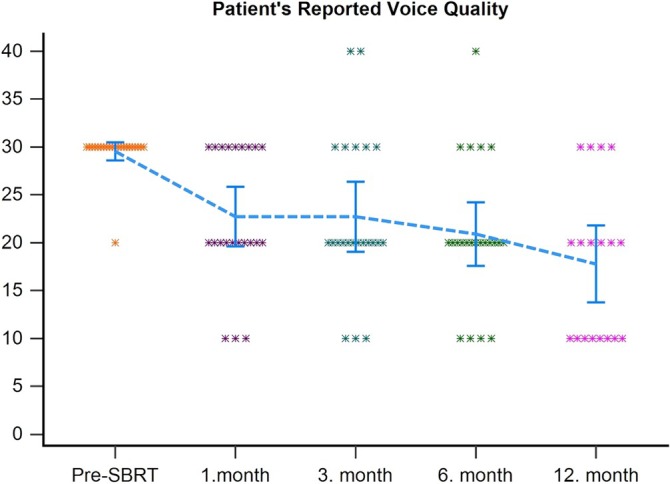
Patient reported voice quality. [Color figure can be viewed at wileyonlinelibrary.com]

Figure 3 demonstrates the change in voice quality noted by patients from the start of the treatment to 12 months after UHRT. The analysis of voice quality scores showed an improvement after UHRT. Statistically significant differences were found between the voice quality before UHRT and the voice quality at the first, third, sixth, and twelfth months after treatment (*p* = 0.03, *p* = 0.01, *p* < 0.01, *p* < 0.01, respectively) (Figure 3).

## 4 Discussion

This study provides valuable insights into glottic UHRT based on real‐world data with long median follow‐up periods. This study's population consisted of unfit patients with many negative characteristics, such as comorbidities and difficulty accessing healthcare services. Additionally, more than half of the patients were active smokers. Nonetheless, we have demonstrated that treating glottic laryngeal cancers with UHRT is safe, even in this patient group. Chung et al. showed that single cord irradiation with conventional fractionation during 29–37 days of treatment time, rather than irradiating the entire larynx, is a feasible treatment strategy [7]. In recent years, there has been a trend in researching shortening treatment times, developing treatments that target smaller areas, or even both without compromising the survival outcomes. Erasmus University Medical Center researchers treated 30 patients using the vocal cord irradiation technique (58.08 Gy/16 fractions) and achieved a 2‐year local control of 100%. Two patients developed in‐field local failure in the long‐term results, so the 5‐year LC rate was 97.1%. The 5‐year OS rate was 80.6%, and the 5‐year laryngectomy‐free survival rate was 98.1%. It has been reported that 4.6% of patients developed laryngeal radionecrosis [8, 9]. Uzel et al. also achieved similar local control with the same treatment protocol [10]. These researchers irradiated the whole cord on the involved side. In our study, one patient (4.5%) had cartilage necrosis and one patient (4.5%) had soft tissue necrosis, similar to the literature data. In contrast, we achieved comparable oncological outcomes (except the OS, possibly due to the characteristics of our patient cohort) and toxicities by irradiating only the lesion with a margin (Table 5).

**TABLE 5:** Summary of studies in the literature on hypofractionated regimens in early‐stage larynx cancer.

References	*n*	RT dose	CTV definition	Technic	Follow‐up (months)	LC	OS	Laryngectomy‐free survival	Necrosis
Erasmus University Medical Center [8, 9]	111	58.08 Gy/16#	Involved cord	IMRT	41	3 years: 99.1% 5 years: 97.1%	5 years: 80.6%	5 years: 98.1%	5%
Uzel et al. [10]	18	57.6–58.08 Gy/15–16#	Involved cord	VMAT	18	2 years: 100%	2 years: 100%		
Schwartz et al. [4]	20	50 Gy/15# (4) 45 Gy/10# (13) 42.5 Gy/5# (3)	GTV + 2 mm cT2 lesions: + IL VC and PGS	Cyberknife	13.4	1 years: 82%	100%	TL: 2 pt	
Sher et al. [11]	25	42.5 Gy/5# (21) 58.08 Gy/16# (4)	iGTV = gross disease	Robotic SBRT	44.4	2 years LFI: 8% (2 pt, in‐field)	4 pt died (2 of them oncologic)	TL: 1 pt	STN: 0
Sanguineti et al. [17, 18]	33	36 Gy/3# 30 Gy/3#	Involved part 1/3 VC next to it	VMAT (97%), IMRT	51.5	4 years: 100%	4 years: 97%		STN: 4 pt CNR: 2 pt (total 18.2%)
Seoul National University [19, 20, 21]	13	Arm I: 47.6–59.5 Gy/17# (7) Arm II: 40.7–55.0 Gy/11# (6)	CTV1: gross disease CTV2: whole larynx	VMAT	Arm I: 37 Arm II: 14.5	Arm I: 100% Arm II: 1 pt in‐field recurrence		TL: 1 pt (Arm II)	CN: 1 pt (Arm II)
Current study	22	35–42.5 Gy/5# (82% 40 Gy)	Gross disease Post VC tumors: + half of the iAC ant VC tumors: + aCom aCom involvement: ant 2 mm of the CL VC	VMAT (86.4%), IMRT	59	5y: 94.7% 5y In‐field: 100%	5y: 68.2%	5y: 89.7%	STN: 1 pt (4.5%) CNR: 1 pt (4.5%)

Abbreviations: #, fractions; aCom, anterior commissure; ant, anterior; CL, contralateral; CNR, chondroradionecrosis; CTV, clinical target volume; GTV, gross tumor volume; iAC, ipsilateral arytenoid cartilage; IL, ipsilateral; IMRT, intensity modulated radiotherapy; LC, local control; LFI, local failure incidence; *n*, number of patient; OS, overall survival; PGS, paraglottic space; post, posterior; pt, patient; RT, radiotherapy; SBRT, stereotactic body radiation therapy; STN, soft tissue necrosis; TL, total laryngectomy; VC, vocal cord; VMAT, volumetric arc therapy; y, year.

The concept of using smaller volumes naturally leads to a reduction in the total number of fractions. Schwartz et al. conducted a phase I study on 20 patients with cTis‐T2N0M0 glottic cancer, investigating three different treatment schedules using robotic SBRT (50 Gy/15 fx, 45 Gy/10 fx, and 42.5 Gy/5 fx). They stated that 42.5 Gy/5 fx is a safe approach and that patients who smoke more than one pack per day have higher toxicity levels [4]. In the same institution, Sher et al. developed a phase II prospective trial to assess the effectiveness of glottic larynx SABR. They utilized a 5‐ or 16‐fraction regimen based on treatment volume and smoking status. Their study revealed two in‐field local recurrences. The 2‐year local failure incidence was 8%, and there were no acute or late grade 3 or higher toxicities in disease‐free patients [11]. In this current study, only three patients had a PTV volume greater than 10 cc, but 59% were active smokers. Unlike the 16‐fraction treatment proposed by Sher et al. for these characteristics, all our patients were treated in 5 fractions with different dose levels. Sher et al. stated that active smokers who undergo SABR have a high risk of soft tissue necrosis or chondronecrosis [11]. Our patient with soft tissue necrosis had never smoked and had a 5.6 cc PTV, which is less than 10 cc. The development of necrosis in this patient, who met the 5‐fraction criteria set by Sher et al. and underwent treatment of 42.5 Gy in 5‐fraction UHRT, suggests that other factors, such as High Dose Rate (HDR), may contribute to the risk of radionecrosis (Table 5).

Vocal cord motion uncertainties have been investigated in some studies. Osman et al. showed that single vocal cord irradiation is not hampered by respiratory motion. Later, they published that using CBCT for daily image guidance in combination with standard mask fixation reduced systematic displacements of the vocal cords to less than 0.2 ± 0.5 mm in all directions before the delivery of each fraction dose [12, 13]. van Asselen et al. showed that swallowing frequency and overall length have little impact on laryngeal intra‐fraction motion. Nevertheless, motion caused by swallowing during the planning CT scan may lead to critical consistent errors. Therefore, swallowing should be prevented during the planning CT scan [14]. In the current study, respiratory motion monitoring was not routinely applied, but daily CBCT imaging was performed. Swallowing was minimized with a hyperextended neck position. Also, with our LINAC‐based treatment, fraction duration is shorter than robotic radiosurgery because of the HDR. Reduced in‐fraction time by HDR may minimize uncertainties related to swallowing. However, it is essential to consider that faster treatment may increase the level and frequency of normal tissue toxicity by enhancing the conversion of sublethal damage to lethal cell damage. The “phantom‐to‐clinic” feasibility study by Ding et al. has confirmed that selective hemilaryngeal IMRT can be comparable to Cyberknife SBRT for sparing doses to normal tissues [15]. Also, phantom measurements showed that the LINAC‐based VMAT plans achieved similar dosimetric endpoints as the Cyberknife planning [16]. Our 5‐year LC with UHRT rate was 94.7%, but the 5‐year larynx preservation rate was 89.7%. The study did not find a significant correlation between increased radiation doses and local control rates. However, higher doses were linked to a loss of organ function. In our study, both patients who received TL were from the 42.5 Gy/5 fraction group. Our treatment field, treatment device, and planning algorithm are similar to those in the trial by Sanguineti et al.; however, the dose definitions differ. They treated the involved part with 36 Gy/3 fractions and the 1/3 portions of the vocal cord next to the involved one with 30 Gy/3 fractions. They pointed out that the 4‐year LC rate was 100%. Four patients experienced soft tissue necrosis, and two patients had cartilage necrosis (a total of 18.2%) [17, 18]. Our analysis indicates that doses exceeding 40 Gy may lead to increased necrosis in LINAC‐based therapy compared to robotic radiosurgery, which we speculate could be attributed to HDR. It is crucial to consider the potential for organ loss despite the high local control rate. The experience of the radiologist, pathologist, radiation oncologist, and head and neck surgeon is extremely important in the post‐treatment management of patients treated with UHRT (Table 5).

Investigators at Seoul National University prescribed different dose levels to the whole larynx and GTV, using a hypofractionated regimen and SIB technique (Arm I: 47.6–59.5 Gy/17 fractions, Arm II: 40.7–55.0 Gy/11 fractions, Arm III: 45 Gy/5 fractions). No patient enrollment data have been shared for the third arm, and the second arm was closed early due to toxicity. A dose of 3.5 Gy per fraction is applicable, but it is important to interpret the results cautiously, as the researchers modified volume definitions in this arm after approximately 5 years. The researchers highlighted the significance of the average hypopharyngeal dose in predicting the occurrence of acute grade ≥ 2 mucositis [19, 20, 21] (Table 5).

The results of the first phase III randomized trial comparing hypofractionated radiotherapy to single cord with TLM are awaited [22]. Again, the phase II randomized trial results comparing 42.5 Gy/5 fractions to vocal cord with whole larynx IMRT at 2.25 Gy per fraction will be guiding [23].

The current study and recent literature suggest that irradiating a smaller volume to a single cord instead of the whole larynx is comparable to oncologic outcomes; however, treatment volumes and doses have not yet been standardized. This study showed similar oncological outcomes with a dose lower than 42.5 Gy in 5 fractions. Additionally, this lower dose seems to be safe regarding radionecrosis in active smokers. This study has several limitations. Some patients were unable to attend their regular follow‐ups for the reasons mentioned. The researchers assessed the voice quality by contacting patients via phone calls or examining visit notes, then scored it accordingly. Therefore, comparing the voice handicap index with those from other studies is not feasible. Second, many treated patients had one or more severe co‐morbidities, and the impact of these on wound healing or toxicity has not been researched. Furthermore, more than 30% of our cohort died within the first 2 years after treatment due to comorbidities or metachron malignancy. If these patients had survived, it is difficult to predict whether a high rate of local control would have been maintained or whether long‐term treatment‐related toxicity would have occurred. Finally, various UHRT doses resulted in inhomogeneity within the patient group.

## 5 Conclusion

In selected patients with early‐stage glottic cancer, single‐cord UHRT appears to be an acceptable treatment for local control. However, in post‐treatment care, it is essential to have an experienced multimodal team. UHRT may produce the possibility of radionecrosis at doses exceeding 41.08 Gy in 5 sessions to PTV D_98_. To demonstrate the role of UHRT in early glottic cancer, results from prospective randomized studies comparing whole larynx radiotherapy and single‐cord radiotherapy are needed.

## Conflicts of Interest

The authors declare no conflicts of interest.

## Supporting information


**Appendix S1.** Supporting Information.

## Data Availability

The data that support the findings of this study are available from the corresponding author upon reasonable request.
