# Supplementary material for: Ultra‐Hypofractionated Radiotherapy for Early‐Stage Glottic Cancer: Real‐World Data From a Single Center
Source: Head Neck. 2025 Apr 1;47(9):2383–91. doi: 10.1002/hed.28139 (PMC12338007; doi:10.1002/hed.28139)
Supplement: Supplementary file 1 — Appendix S1. Supporting Information. [file HED-47-2383-s001.docx]

| **Patient No** | **Suspicion of Recurrence (months)** | **First Biopsy** | **Salvage Treatment** | **Final Pathology** |
| --- | --- | --- | --- | --- |
| 4 | Radiological (16) | SCC | TL + BLNDx | %10 SCC, %90 soft tissue necrosis |
| 16 | Radiological (8) | SCC | Cordectomy | No signs of tumor, only fibrosis |
| 19 | Clinical, shortness of breath (6) | chronic inflammation | - | - |
| 21 | Clinical, hoarseness (4) | chronic inflammation | - | - |
| 22 | Radiological (9) | SCC | TL + BLNDx | No signs of tumor, only chondronecrosis and fibrosis |
| SCC: Squamous Cell Carcinoma; TL + BLNDx: Total Laryngectomy + Bilateral Neck Dissection | | | | |

Despite showing  complete response to treatment, Patient number 4 was suspected of tumor recurrence 16 months later due to diffuse laryngeal edema detected in endoscopic and radiological examinations. The disease initially involved the right vocal cord, but the biopsy from the left subglottic area showed SCC, with no tumor tissue found in the irradiated glottic region. The patient subsequently underwent a total laryngectomy and bilateral neck dissection (TL). The pathology report revealed 90% necrosis and 10% SCC in the removed tissue. Patient 22 had a history of cordectomy due to dysplasia ten years before UHRT. A biopsy was performed nine months after the treatment, as the imaging results indicated a possible recurrence. The biopsy revealed SCC, leading to the patient undergoing TL. However, no tumoral tissue was found in the postoperative specimen; only necrosis and fibrotic changes were present. Patient 16 was found to have soft tissue involving the vocal cord and ventricular band during the eight months of follow-up, and the pathology report was SCC. The patient underwent a cordectomy in the 10^th^ month. No tumoral tissue was found in the suspected area or the other areas. In patients 19 and 21, biopsies were taken in the sixth and fourth months due to clinical suspicion of recurrence (complaints of shortness of breath and hoarseness). The biopsies were reported as showing chronic inflammatory changes. The 19th patient, whose symptoms completely disappeared after medical treatment, passed away 18 months after UHRT due to cardiac reasons. In contrast, the other patient leads a healthy life without symptoms. All other patients had no suspicion of primary recurrence, neck lymph node metastasis, or distant metastasis.
